# A Claim in Respect of Tetanus

**Published:** 1911-09-23

**Authors:** 


### Medico-Legal Points.

### A CLAIM IN RESPECT OF TETANUS.

In a case at Edinburgh (Turner *v.* Wilson, June 13), a claim was made by the widow of a gardener who died of tetanus on September 1, 1910. Reid lived in his own house, which was not on the property of the employer, and had a garden of his own which he worked. On the morning of Sunday, August 21, he had been in his master's garden. After returning to his own house he proceeded to clean his boots without taking them off, and while doing so he tore his right thumb under the nail against a lacing hook of one of his boots. Four days afterwards tetanus set in, and he died from exhaustion and heart failure as the result of tetanus. There was no evidence of how or when the dirt under the nail was obtained, but looking to the nature of the employment, and the fact that the bacillus of tetanus is generally found in manure and leaf mould, the Sheriff Substitute held that it was reasonable to hold that it was obtained by Reid when working in his master's garden, but on the ground that the accident did not arise out of and in the course of his employment the Sheriff Substitute refused Mrs. Reid and her children compensation.

The Division affirmed the decision of the Sheriff Substitute and found the appellant liable in expenses.

The Lord Justice Clerk said the facts did not warrant a claim for compensation. The deceased, after leaving his master's premises for his own house was no longer in his master's employment. There was then no relation between him and his employer. He was at liberty to attend to his affairs, and was not when he met with the accident acting in the course of his employment. It was maintained that the bacillus got into the man's thumb while he was working in the employer's garden, and that accordingly he must be held to have been injured in the course of his employment. Assuming that such a contention could be held to be logically sound, before it could be given effect to it must be proved as a necessary inference that the bacilius had become attached to the deceased's body while he was doing his master's work. The question was not whether it was a reasonable inference; it was whether it was the only reasonable inference. There was no ground for holding that the contracting of the bacillus in the master's garden was the exclusive inference to be drawn from the facts. It was just as reasonable to hold that he contracted bacillus in his own garden.

